# Potential Health Benefits of Dietary Tree Nut and Peanut Enrichment in Kidney Transplant Recipients—An In-Depth Narrative Review and Considerations for Future Research

**DOI:** 10.3390/nu17152419

**Published:** 2025-07-24

**Authors:** Daan Kremer, Fabian A. Vogelpohl, Yvonne van der Veen, Caecilia S. E. Doorenbos, Manuela Yepes-Calderón, Tim J. Knobbe, Adrian Post, Eva Corpeleijn, Gerjan Navis, Stefan P. Berger, Stephan J. L. Bakker

**Affiliations:** 1 Division of Nephrology, Department of Internal Medicine, University of Groningen, University Medical Center Groningen, Post Box 30.001, 9700 RB Groningen, The Netherlands; f.a.vogelpohl@umcg.nl (F.A.V.); y.van.der.veen@umcg.nl (Y.v.d.V.); c.s.e.doorenbos@umcg.nl (C.S.E.D.); m.yepes.calderon@umcg.nl (M.Y.-C.); a.post01@umcg.nl (A.P.); g.j.navis@umcg.nl (G.N.); s.p.berger@umcg.nl (S.P.B.); s.j.l.bakker@umcg.nl (S.J.L.B.); 2 Division of Nursing Science, Department of Health Sciences, University of Groningen, University Medical Center Groningen, 9700 RB Groningen, The Netherlands; 3 Department of Dietetics, University of Groningen, University Medical Center Groningen, 9700 RB Groningen, The Netherlands; 4 Department of Epidemiology, University of Groningen, University Medical Center Groningen, 9700 RB Groningen, The Netherlands; e.corpeleijn@umcg.nl

**Keywords:** dietary intervention, inflammation, fibrosis, aging, micro-nutrients, oxidative stress, nuts, kidney transplant recipients, very long-chain saturated fatty acids

## Abstract

Kidney transplant recipients face a substantial burden of premature mortality and morbidity, primarily due to persistent inflammation, cardiovascular risk, and nutritional deficiencies. Traditional nutritional interventions in this population have either focused on supplementing individual nutrients—often with limited efficacy—or required comprehensive dietary overhauls that compromise patient adherence. In this narrative review, we explore the rationale for dietary nut enrichment as a feasible, multi-nutrient strategy tailored to the needs of kidney transplant recipients. Nuts, including peanuts and tree nuts with no added salt, sugar, or oil, are rich in beneficial fats, proteins, vitamins, minerals, and bioactive compounds. We summarize the multiple post-transplant challenges—including obesity, sarcopenia, dyslipidemia, hypertension, immunological dysfunction, and chronic inflammation—and discuss how nut consumption may mitigate these issues through mechanisms involving improved micro-nutrient intake (e.g., magnesium, potassium, selenium), lipid profile modulation, endothelial function, immune support, and gut microbiota health. Additionally, we highlight the scarcity of randomized controlled trials in high-risk populations such as kidney transplant recipients and make the case for studying this group as a model for investigating the clinical efficacy of nuts as a nutritional intervention. We also consider practical aspects for future clinical trials, including the choice of study population, intervention design, duration, nut type, dosage, and primary outcome measures such as systemic inflammation. Finally, potential risks such as nut allergies and oxalate or mycotoxin exposure are addressed. Altogether, this review proposes dietary nut enrichment as a promising, simple, and sustainable multi-nutrient approach to support cardiometabolic and immune health in kidney transplant recipients, warranting formal investigation in clinical trials.

## 1. Introduction

### 1.1. Inflammation and Metabolic Concerns in Kidney Transplant Recipients

Even after successful transplantation, there is excess morbidity and premature mortality among kidney transplant recipients compared to the general population [1]. Inflammation and cardiovascular risk are considered important underlying causes of this post-transplant burden [2,3,4,5,6]. The pro-inflammatory state after kidney transplantation can partly be explained by concomitant conditions such as hyperglycemia, dyslipidemia, hypertension, excess body fat, history of kidney failure and comorbidities [3,5,6,7]. Oxidative stress due to ischemia–reperfusion injury and post-transplantation factors such as nutritional concerns and elevated advanced glycation end-products further contribute to renal and systemic inflammation [8,9,10,11]. Next to these non-immunological factors, kidney transplant recipients suffer from an inflammatory burden due to immunological responses towards the kidney allograft [1,3]. The clinical challenge is that the adaptive immune system must be suppressed to prevent graft rejection, while maintaining sufficient adaptive and innate immune responses to combat infections. As a result, there is an urgent clinical need to alleviate the burdens of inflammation and cardiovascular risk in kidney transplant recipients. Dietary enrichment with nuts in healthy individuals as well as in patients with chronic metabolic conditions and metabolic syndrome, which is highly prevalent in kidney transplant recipients [12], has been shown to effectively decrease multiple markers of inflammation and oxidative stress [13,14]. Therefore, we propose dietary nut enrichment as a potential dietary adjuvant that may potentially yield benefit for several of these burdens.

### 1.2. Definition of Nuts

Considerations regarding the use of different definitions of nuts and seeds are excellently summarized elsewhere [15]. According to their botanical definition, nuts are “dry one-seeded fruit with an extremely hard pericarp (outer layer of the ovary wall)” [15]. However, as this definition is usually not considered for analyses of health outcomes and by dietary guidelines, for the current review, nuts are defined according to the proposed ‘nutritional research definition’, which groups nuts according to their nutritional profile, including almonds, Brazil nuts, cashews, hazelnuts, macadamias, peanuts, pecans, pine nuts, pistachios and walnuts [15]. The composition of nuts included in this definition differs between types, but they generally have low water and carbohydrate content, yet high concentrations of proteins, mono- and polyunsaturated fats, fiber, micro-nutrients and phytosterols [15]. An overview of the nutritional content of nuts is provided in Table 1. This explains why peanuts are included, because peanuts have a similar nutrient profile to other nuts. In contrast, chestnuts are not included in this definition considering their high carbohydrate and water content, and coconuts are excluded because of their high saturated fat content [15]. When referring to nutrient content of mixed nuts in this manuscript, we refer to a mix of nuts that is often consumed and is composed of peanuts (48.2%), cashew nuts (24.5%), walnuts (11.6%), blanched almonds (4.2%), hazelnuts (4.1%), macadamia nuts (2.9%), pecan nuts (2.4%) and Brazil nuts (2.2%), according to the Dutch Food Composition Table [16]. Notably, we refer to nuts with no added salt, sugar or oil.

### 1.3. Rationale for Studying Anti-Inflammatory and Cardiometabolic Effects of Nuts in Kidney Transplantation

To date, most previous studies assessed potential effects of dietary nut enrichment in low-risk populations, such as in study participants without any disease, or participants with hypertension, hyperlipidemia, or metabolic syndrome [18,19,20,21,22,23]. Meta-analyses of randomized controlled trials have shown beneficial effects of nut consumption on blood pressure, lipid status and endothelial function [18,19,24]. Randomized controlled trials on this topic in high-risk populations such as kidney transplant recipients are lacking [24]. The suggested effects of nuts—including improvements in inflammation, fibrosis, endothelial function, blood pressure, lipid profile, oxidative stress, and nutritional deficiencies [14,22,23]—are particularly relevant in kidney transplant recipients, and these measures are consistently associated with clinical outcome in kidney transplant recipients [1,3,4,6,24,25,26,27]. As a result, potential effect sizes are larger, and the number of patients needed to confirm treatment effects is lower compared to low-risk populations. Kidney transplant recipients may thus serve as a model population to assess proof of principle.

**Figure 1:**
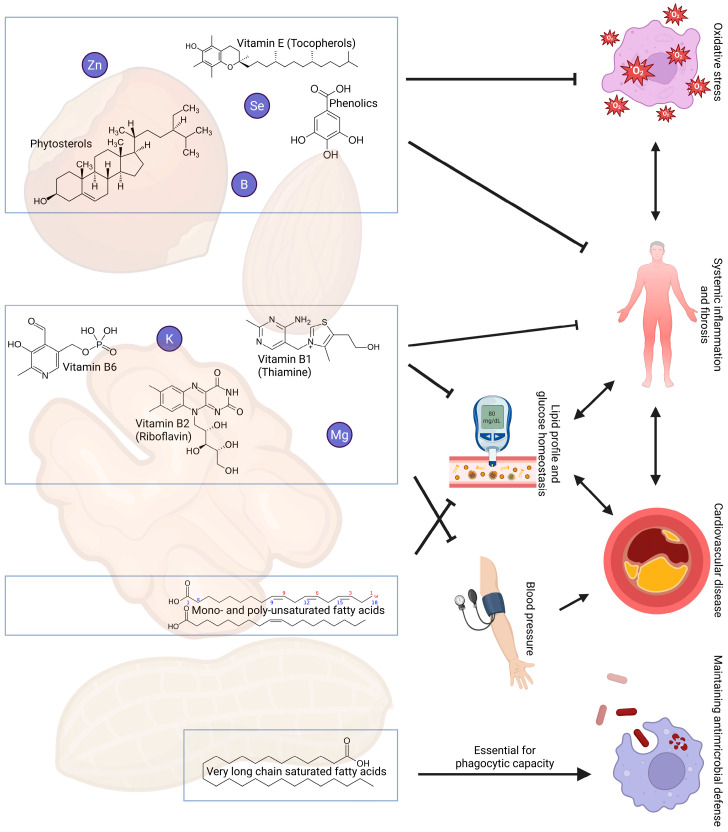
Overview of selected nutrients present in nuts that have suggested beneficial effects on cardiovascular risk, inflammation and the immune system in kidney transplant recipients. Abbreviations: B, boron; K, potassium; Mg, magnesium; Se, selenium; Zn, zinc.

In this narrative review, we therefore provide a rationale for a randomized controlled trial studying the effects of dietary nut enrichment in kidney transplant recipients. To do so, we will summarize nutritional and metabolic burdens that kidney transplant recipients face, alongside an explanation of how dietary nut enrichment may alleviate these burdens as a dietary adjuvant. A visual overview is presented in Figure 1. We note that the aim of the current manuscript is not to focus in detail on underlying molecular mechanisms, as these are too diverse and complex to describe in one review. The review ends with considerations for a future randomized controlled trial with mixed unsalted nut intervention in kidney transplant recipients.

## 2. Dietary Nut Enrichment as a Simple, Feasible Multi-Nutrient Intervention

Notably, current treatment guidelines do not provide dietary recommendations for kidney transplant recipients at all. Instead, they focus on the management of metabolic derangements such as hypertension, dyslipidemia, obesity and cardiovascular disease [28]. However, nutritional intervention is a promising strategy to alleviate the nutritional and cardiometabolic problems that are highly prevalent among kidney transplant recipients [29].

Simplicity, feasibility and effectiveness are prerequisites of a successful nutritional intervention. Most previous nutritional trials aimed to improve health by either supplementing a single nutrient, which may have limited effect, or by radically altering lifestyle and diet, which limits (sustainable) compliance. Both approaches have had limited success [24,30,31,32]. A promising alternative strategy is a multi-nutrient intervention [33,34]. Rather than supplementation of nutrients through pills, we propose nuts as a promising multi-nutrient intervention that is particularly relevant in the kidney transplant recipient population. Dietary nut enrichment in kidney transplant recipients is attractive as it is simpler and more feasible compared to extensive dietary or lifestyle regimes due to their high micro-nutrient content (Table 1). Dietary nut enrichment may therefore correct multiple micro-nutrient deficiencies that are prevalent in kidney transplant recipients and that are associated with adverse clinical outcome in various populations, including kidney transplant recipients [31,32,35,36,37,38,39,40]. In other populations, the beneficial effects of nut interventions on cardiovascular disease and mortality have been shown [20,21,41]. Moreover, nuts are generally included in the items considered to determine Mediterranean diet adherence, which is consistently associated with improved outcomes in many populations, including kidney transplant recipients [42]. The suggested beneficial cardiovascular and metabolic effects of nuts are attributed to several distinct mechanisms which will be described in detail in the next section. In this section, we will also explain why these nutritional and metabolic concerns are particularly relevant to kidney transplant recipients.

### 2.1. Body Composition and Nutritional Status

Post-transplant weight gain is common, largely attributed to lifestyle factors and the use of immunosuppression, including corticosteroids [43,44]. As a result, abdominal obesity occurs in approximately 50% of kidney transplant recipients. While there is some degree of muscle mass improvement after transplantation, relative sarcopenia persists [45]. These post-transplant changes in body composition are cause for concern, because both adiposity and low muscle mass are associated with a markedly increased risk of mortality [46,47]. Dietary nut enrichment may be a strategy to alleviate the burdens of obesity and protein malnutrition in kidney transplant recipients.

#### 2.1.1. Obesity

Contrary to popular belief, intake of nuts does not lead to weight gain, regardless of dietary substitution instructions [48,49,50]. In fact, high nut intake is associated with a lower risk of obesity [48]. Specifically in kidney transplant recipients, a higher adherence to a diet rich in nuts was observed to be associated with lower fat mass [51]. There are several potential explanations for these observations, including the relatively high satiety index of nuts and a phenomenon called energy compensation (short-term effects of compensation in next meals, and long-term effects that may last for weeks or months, regardless of dietary substitution analyses) [52]. Moreover, although nuts are calorie-dense, with a handful of nuts accounting for ~200 kCal (i.e., ~800 kJ), recent evidence suggests that not all energy within nuts is metabolizable, which further explains the lack of obesogenic effects [53]. Therefore, despite their relatively high calorie-density and fat content, dietary nut enrichment in kidney transplant recipients does very likely not lead to an increase in fat mass.

#### 2.1.2. Protein Malnutrition

Nuts may also improve nutritional status as they are a source of protein. Before transplantation, patients with kidney failure are generally advised to adhere to a low-protein and low-salt diet. While the latter is still advisable after transplantation, the first may no longer be advisable. Yet, a low-protein diet may still be a self-imposed restriction stemming from the pre-transplant advices. Although there is a lack of guidelines for protein intake in kidney transplant recipients, higher protein intake is consistently associated with improved clinical outcomes in this population, as well as less fatigue and higher health-related quality of life [54,55,56]. This association between protein intake and improved clinical outcomes appears to be mediated by muscle mass [57]. One handful of mixed nuts contains approximately 6 g of protein, which is a meaningful contribution against the protein malnutrition that is frequently observed among kidney transplant recipients [16,54]. The biological value of nut proteins represented by the Protein Digestibility-Corrected Amino Acid Score (PDCAAS), which assess both human amino acid requirements of humans and digestibility, ranges between 44% for almonds and 90% for cashews, with most nuts having a PDCAAS of roughly 60–70% [58]. On the first look, these numbers might sound somewhat inferior compared to those of meat, which has a PDCAAS of 92–95% depending on the animal source [58]; however, integrating different nuts, seeds, legumes within a diet, even if the diet is vegetarian, can be sufficient to reach full amino acid requirements in adults, given sufficient energy intake and food variation [59]. Dietary nut enrichment within an omnivorous diet is therefore well suited to increase protein intake and prevent protein malnutrition in kidney transplant recipients.

### 2.2. Cardiovascular Health—Blood Pressure and Vascular Calcification

Although kidney transplantation recipients show decreased cardiovascular events after successful transplantation compared patients receiving dialysis, kidney transplant recipients still display 50 times more annual cardiovascular events and show a 10 times higher cardiac death rate compared to the general population [60]. Dietary nut enrichment might be a valuable measure to reduce these risks as tree nut and peanut consumption showed to decrease the risk of cardiovascular disease and specifically of coronary heart disease by 21% and 29%, respectively, per 28 g/day increase in nut intake [21]. Two major, interconnected risk factors for cardiovascular events in kidney transplant recipients are hypertension and vascular calcification [60]. Hypertension occurs in 50% to 85% of kidney transplant recipients [61]. Prolonged hypertension can damage end-organs and cause arterial stiffening and vascular calcification [62,63]. Indeed, in kidney transplant recipients, hypertension is consistently associated with worse graft survival and patient survival [61,64,65,66,67,68]. As a result, there is a clinical need to improve post-transplant hypertension control and to mitigate arterial stiffness and vascular calcification [69,70,71]. Dietary nut enrichment (without added sugar, salt or oil) may help to alleviate these factors, thanks to beneficial effects on endothelial function and blood pressure [18,22,23]. A recent observational study further specifically showed that higher nut and seed intake was associated with a lower risk for abdominal aortic calcification [72]. Although nut intervention studies that could verify this beneficial effect on vascular calcification, especially in kidney transplant recipients, are lacking to date, dietary nut enrichment has the potential to decrease hypertension, vascular calcification and cardiovascular events in this vulnerable patient group.

#### 2.2.1. Magnesium

The beneficial effects of nuts on these cardiovascular factors are partly attributable to improved magnesium status. Magnesium may lower blood pressure and prevent vascular calcification through various physiological mechanisms [73,74,75]. Indeed, hypomagnesemia is consistently linked to many chronic diseases, including hypertension and cardiovascular disease [76], while higher magnesium intake associates with lower cardiovascular risk [77]. Magnesium intake is generally lower than the Dietary Reference Intake (DRI) of 310 to 420 mg/day [78], in particular in kidney transplant recipients [27]. Next to a suboptimal dietary magnesium intake, magnesium status in the kidney transplant population is further compromised by the frequent use of calcineurin inhibitors, which inhibit tubular magnesium reabsorption [79], and the frequent use of proton pump inhibitors that impair magnesium absorption from the gut [27]. Dietary nut enrichment may significantly improve magnesium status, as ingestion of 50 g of mixed nuts accounts for approximately 30% of the DRI of magnesium [16,78]. It appears plausible that such increments in magnesium intake can exert beneficial effects on inflammation and cardiometabolic risk which may be relevant for kidney transplant recipients, although interventional studies are needed to prove such effects [80,81,82].

#### 2.2.2. Potassium

Next to magnesium, the beneficial effects of nuts on the vasculature may be explained by potassium. Although high potassium intakes have long been considered detrimental within the field of nephrology because of presumed risks of hyperkalemia, this paradigm has shifted in recent years [83]. The potential beneficial effects of potassium-rich food are increasingly recognized among patients with kidney disease [84]. Exact underlying mechanisms are unclear, but preclinical studies suggest that the protective effect of potassium may be attributable to upregulation of renal kinins and concomitant decreases in blood pressure [85]. Yet, regardless of the underlying mechanisms, the associations of higher potassium intake with improved cardiometabolic profiles are consistent in humans [86]. Studies in kidney transplant recipients also showed that higher urinary potassium excretion, reflecting higher intake, was associated with a lower risk of both graft failure and mortality, independent of potential confounders [87]. Nuts are particularly rich in potassium. Dietary enrichment of 50 g mixed nuts accounts for ~20% of the recommended daily intake [16], and may thus substantially help to increase potassium intake in the kidney transplant population [87].

### 2.3. Cardiovascular Health—Lipid Status

Next to direct effects on the vasculature, nut intake may lower cardiovascular risk through improving lipid status. In kidney transplant recipients, dyslipidemia is highly prevalent, which is largely attributed to comorbidities, lifestyle factors and corticosteroid use as part of immunosuppressive therapy [88,89,90]. Dyslipidemia is a major risk factor for adverse outcome in the general population and among kidney transplant recipients [88,89,91]. Because of its clinical relevance, treatment of dyslipidemia by statins is endorsed by current post-transplant treatment guidelines [92]. However, statins should be used with caution and there is a need for safe alternative strategies to improve lipid status after kidney transplantation [89]. Dietary nut enrichment appears an attractive strategy. Multiple studies have shown that nut intake lowers total cholesterol, LDL cholesterol, apolipoprotein B and triglycerides concentrations, although evidence quality varies [19,22]. The effects on lipid status are largely attributable to the favorable composition of nuts. Nuts have high lipid content ranging from 42 to 76 g/100 g. Generally, this lipid content is mainly consisting of monounsaturated fatty acids and/or polyunsaturated fatty acids, depending on the type of nut [93,94]. Oleic acid (C_18:1_) is the main constituent of monounsaturated fatty acids in nuts, whereas alpha-linolenic acid (C_18:3_) and linoleic acid (C_18:2_) are the major omega-3 and omega-6 polyunsaturated fatty acids constituents [16,95]. Indeed, higher intake of these fatty acids—in particular alpha-linolenic acid—is associated with lower risk of cardiovascular disease and mortality [95,96], likely in part through attenuation of inflammation [97]. Similar beneficial effects, primarily of omega-3 fatty acids, have also been proposed in kidney transplant recipients [98,99].

Moreover, phytosterols such as β-sitosterol, are present in relatively high concentrations in nuts [100]. Although phytosterol content differs significantly between nut types, a 30 g serving of nuts generally contains between 30 and 100 mg of phytosterols, most of which is β-sitosterol [101]. This is a significant contribution, considering that natural dietary intake varies from about 167 to 437 mg/day, while intakes of >1500 mg/day are advised in various health recommendations [102,103]. Although studies in the kidney transplant population are lacking, there is preclinical evidence that β-sitosterol has anti-atherosclerotic, triglyceride-lowering, anti-inflammatory and anti-oxidative properties [104,105,106,107]. Although intervention studies in humans are lacking, phytosterols are associated with lower LDL cholesterol [108]. Taken together, these suggested beneficial effects may contribute to improving lipid status and cardiovascular health in kidney transplant recipients.

### 2.4. Immunological Status

Circulating concentrations of very long-chain saturated fatty acids (VLSFA) including arachidonic acid, behenic acid and lignoceric acid are lower in kidney transplant recipients, compared to healthy controls [12]. Notably, these low levels are associated with a higher risk of (infectious) mortality [12]. A comparable beneficial effect of VLSFA on infectious mortality has further been reported in the general population as well [109], implying that these beneficial effects of VLSFA are not limited to immunocompromised individuals. Particularly peanuts are rich in behenic acid and lignoceric acid [110]. Interestingly, it has been shown that intake of peanuts is associated with a decreased risk of all-cause mortality and mortality from respiratory and infectious disease in the general population [21]. We therefore speculate that VLSFA contained in peanuts are a driver for the beneficial associations observed between peanut intake and clinical outcomes in various observational studies. Although these findings are promising, in many ways epidemiological findings have preceded our knowledge regarding the influence of VLSFA on health outcomes, especially regarding infection susceptibility. Therefore, a mechanistic understanding of these observations is highly needed. It has been shown that VLSFA-sphingolipids are essential for various functions of the innate immune system, including maturation of macrophage phagosomes [111]; efficient phagocytosis by macrophages [112]; neutrophil migration [113]; IL-6 secretion [114]; and IL-10 signaling [115]. Overall, these findings suggest that VLSFA play a broad and crucial role in the clearance of pathogens by the innate immune system.

Next to very long-chain saturated fatty acids, peanuts are rich in L-arginine, a semi-essential amino acid. According to the USDA National Nutrient Database, raw peanuts contain approximately 3.09 g of L-arginine per 100 g, positioning peanuts among the top dietary sources of L-arginine [116]. Research in septic mice indicates that supplementation with L-arginine can augment the phagocytic capacity of macrophages [117]. This immunomodulatory effect is largely attributed to L-arginine’s role as a precursor for nitric oxide (NO) synthesis. Nitric oxide is a key signaling molecule involved in various aspects of immune function, including the acidification of phagosomes, the generation of reactive oxygen species (ROS), and the regulation of NO consumption during microbial killing [118,119,120].

Several other micro-nutrients that were previously mentioned, including magnesium, may also have beneficial immunological effects, including roles in activating or regulating leukocyte and macrophage activation, release of inflammatory cytokines and acute phase proteins, and free radical production [121,122,123]. Summarizing, due to their content of immune-stimulating nutrients, nuts and peanuts in particular are an attractive hypothetical target to improve innate immunity in kidney transplant recipients [12,124,125].

### 2.5. Gut Health

Dietary fiber consists of a group of non-digestible carbohydrates, with many suggested beneficial health effects, including a lower risk of atherosclerosis and cardiovascular disease [126]. Although evidence in kidney transplant recipients is lacking, the beneficial effects of dietary fiber on the microbiome, reduced inflammation and oxidative stress suggest that improving fiber intake may also yield benefits in this population [127,128]. Peanuts in particular rich in fibers, containing both soluble and insoluble fiber, with insoluble fibers including cellulose and lignin being more abundant, especially in the peanut skin [129,130]. One handful of unsalted mixed nuts contains approximately 2 g of fibers [16]. Although the effect sizes of 2 g of increment in fiber intake may be limited [131], this amount does translate into a substantial proportion of the dietary reference intake [132]. Next to fiber alone, nuts may have modulatory effects on the gut microbiota through other mechanisms including polyphenols, although results are inconsistent [133]. Nevertheless, any improvements in gut health after transplantation appears very relevant for clinical outcome [134,135], raising the hypothesis that this may be another pathway through which kidney transplant recipients may benefit from nuts.

### 2.6. Inflammation

Chronic inflammation is tightly related to the etiology of disease across the life span in many ways [136]. Kidney transplant recipients generally suffer from a systemic pro-inflammatory state with consequent fibrosis, with suggested detrimental effects on graft and patient prognosis [4,25,137,138]. In kidney transplant recipients, inflammation and fibrosis may exert their detrimental effects through increased risk of new-onset diabetes after transplantation, accumulating kidney graft damage, increased vascular calcification and atherosclerosis [4,6,7,25,139,140,141,142,143,144]. Although evidence is inconclusive, studies suggest that dietary nut enrichment improves inflammation and oxidative stress [14]. There are several biological explanations for the suggested beneficial effects of nuts on inflammation. They include the previously mentioned factors such as cardiovascular risk, obesity, immunological state and other comorbidities, because they frequently coincide with increased levels of chronic, low-grade inflammation [136,145,146]. In addition to these factors, micro-nutrient deficiencies may be an important cause of inflammation, as explained hereafter.

The high prevalence of magnesium deficiency in kidney transplant recipients was described previously in this review. However, in addition to direct effects on the vasculature, other consequences of low magnesium status include chronic low-grade inflammation and oxidative stress: a notion that was already coined over 90 years ago [147]. Next to magnesium, nuts contain significant amounts of tocopherols (e.g., ~15% of recommended daily intake of α-tocopherol equivalents per portion of pistachios or walnuts [148]), carotenoids (mainly lutein/zeaxanthin [148,149]), phenolics (e.g., flavonoids, resveratrol, exact content varies largely depending on the type of nut [149,150,151,152,153]), B-vitamins (e.g., vitamins thiamine/B1, riboflavin/B2 and total vitamin B6 [148]). Polyphenols including anthocyanins, flavonoids, lignans, naphthoquinones, phenolic acids, proanthocyanidins, stilbenes and tannins have been identified in nuts [154,155]. Their suggested beneficial health effects are primarily attributed to inhibition of oxidative stress, thus preventing lipid oxidation, protein oxidation and DNA damage [154,156]. Furthermore, trace elements including boron (50 g of nuts is sufficient to meet the minimal safe mean population dietary boron intake of 1000 µg suggested by the WHO [157,158,159,160,161,162,163]), selenium (50 g of mixed nuts contains approximately half of the recommended daily allowance, particularly high concentrations in Brazil nuts [15,164]), and zinc (a handful of mixed nuts contains ~10% of zinc reference intake recommendations [15,165]) are all relatively abundant in nuts.

While the structures and biological functions differ between these nutrients, they all have suggested anti-inflammatory and anti-oxidative effects [40,153,166,167,168,169,170,171,172,173,174,175,176,177]. Notably, tocopherols [178], boron [35], selenium [179], vitamin B6 [180] are all associated with prognosis in kidney transplant recipients. Other nutrients including phenolics, zinc and thiamine have not been studied in the kidney transplantation, but are relevant in kidney disease [10,166,167,180,181,182,183,184,185,186,187,188,189,190]. All in all, it is plausible that nuts may exert beneficial anti-inflammatory effects through myriad pathways.

## 3. Potential Risks or Concerns of Dietary Nut Enrichment

### 3.1. Allergies

Unfortunately, epidemiological studies on food allergies in kidney transplant recipients are lacking, the prevalence of nut allergies is estimated to be 1 to 2% in the Western world [191] and because of the chronic use of immunosuppressive medication, this is likely lower than in kidney transplant recipients, but assessment of the actual prevalence requires further investigation. Many individuals with this allergy report serious detrimental health effects after ingestion of the allergen. Clearly, it is important to consider any potential allergies prior to nut consumption, and any nut allergy should be an exclusion criterion from any nut intervention study.

### 3.2. Oxalate

Oxalate is a product of ascorbic acid metabolism in humans, and it naturally occurs in nuts. Oxalate nephropathy is a rare form of kidney injury from calcium oxalate crystal deposition in the kidney parenchyma. Although nuts generally appear not to cause hyperoxaluria, there are some case reports of excessive tree nut intake leading to oxalate nephropathy [192]. However, notably, in these cases the nut intake far exceeded one or two handfuls per day, and oxalate appears a limited concern when ingesting relatively small amounts of nuts, considering that the oxalate content of nuts is lower than spinach or tea [193,194].

### 3.3. Mycotoxins

Mycotoxins are metabolites of fungi and receive increasing scientific attention as they appear a threat to food quality and human health [195]. A recent study found low levels of mycotoxins in almonds, peanuts, walnuts, hazelnuts, pecan nuts, cashews, Brazil nuts and pine nuts, and indicated that there is no cause for concern for individuals exposed to mycotoxins through consumption of nut products [196]. However, mycotoxins are an area of ongoing investigation [197]. It is plausible that mycotoxins may be of particular relevance in kidney transplant recipients, given their diminished kidney function and immunosuppressed state, but epidemiological evidence is lacking. Common thermal processing methods like roasting are generally considered ineffective for significantly reducing mycotoxin levels in peanuts [198]. However, irradiation and other techniques are being explored to lower the risk of detrimental effects of mycotoxins [197,199]. Although generally, nuts with mycotoxin levels exceeding limits are removed from the food supply chain [200], it would be interesting for a future intervention trial to explore potential effects of dietary nut enrichment on mycotoxin exposure.

### 3.4. Phosphate/Phosphorus

Hyperphosphatemia is associated with worse clinical outcomes in multiple populations, including kidney transplant recipients [201,202]. However, hyperphosphatemia is generally not a major clinical problem after kidney transplantation. Instead, hypophosphatemia is generally a larger clinical problem in this population [203]. Even if we consider hyperphosphatemia as a potential concern, it is unlikely that the phosphorus/phosphate content (<250 mg per 50 g of mixed nuts [15]) in nuts is problematic. Moreover, even in patients with (severe) chronic kidney disease, phosphate intake is not tightly linked with circulating phosphate or hyperphosphatemia [204,205]. Thus, hyperphosphatemia and the associations with impaired outcomes in kidney transplant recipients are likely the result from non-dietary factors, such as hyperparathyroidism [203].

## 4. Discussion

In this narrative review, we showed that dietary enrichment of tree nuts and peanuts in kidney transplant recipients can potentially improve multiple health concerns in this vulnerable population, specifically overweight, protein malnutrition, cardiovascular disease, micro-nutrient deficiencies, inflammation, immunoregulation and dysbiosis. Based on this, we now reflect on current and advised nut intake and how tree nuts and peanuts can be clinically implemented in the form of a dietary multi-nutrient intervention.

### 4.1. Current and Advised Nut Intake

Because of its numerous suggested health benefits, nut intake is promoted in dietary guidelines for the general population. Although recommended serving sizes vary, most guidelines suggest a daily intake of 15 to 30 g of nuts [206]. Population consumption data suggests that most individuals fail to meet current recommendations for nut intake [207]. In fact, among all assessed major foods and nutrients in the Global Burden of Disease Study, the gap between observed global intake and optimal intake was largest for nuts, with an observed consumption of only 12% of the optimal intake defined as >21 g of nuts per day [207]. The recommendations for nut intake are even less frequently met by kidney transplant recipients, with only 7%, 4%, and 1% of kidney transplant recipients consuming at least 15, 21, or 30 g of nuts per day, respectively (unpublished data, derived from Kremer et al. [35]). Potential barriers to meeting these recommendations include confusion regarding the effects of nut consumption on body weight, concerns regarding the high fat content of nuts, or concerns about cost [208]. Conversely, higher levels of education and income, as well as a generally healthier lifestyle, are associated with higher nut intakes [208]. Health professionals can play a crucial role in promoting nut consumption; however, research indicates that many health professionals are not familiar with the plethora of health benefits accompanying adequate nut consumption [208]. An intervention study showing health benefits of nut consumption is needed to elucidate the potential role of dietary nut enrichment in clinical care and may help to remove any misunderstandings regarding nut intake. In the following sections, we address considerations for future studies assessing such effects.

### 4.2. Considerations for Study Population

As previously explained in detail, kidney transplant recipients appear to be an excellent study population for both epistemological reasons (suggested large effect size] and clinical reasons (burdened with comorbidities, inflammation, oxidative stress, nutritional concerns including nutrient deficiencies, etc.). To avoid transplant complications or other clinical events unrelated to the intervention blurring the potential effects of the intervention, we suggest including clinically stable kidney transplant recipients at least one year after transplantation. From this point onwards, patients generally have a stable immunosuppressive regimen. Clearly, patients with nut allergies should be excluded from the study, but we suggest keeping other eligibility criteria lean to facilitate extrapolation of our findings to the broader kidney transplant population, and potentially to other high-risk populations suffering from similar inflammatory and cardiometabolic burdens.

### 4.3. Considerations for Study Design

Multiple study designs may be considered in future research. A cross-over study is appealing considering the high power. However, this design comes at a risk of carry-over effects, particularly if the nut intervention does indeed treat nutrient deficiencies. Alternatively, studies assessing health effects of nuts may employ a parallel-group design, where a larger sample size is likely needed but carry-over effects are not present. A parallel-group design may also more easily detect prolonged/delayed beneficial effects of a nut intervention, because patients do not switch treatment midway through the study. Additionally, a simple parallel-group design allows for a pragmatic approach where patients need less study visits compared to a cross-over study. Whenever possible, site visits can easily be combined with appointments for clinical care. A parallel-group design, because of its simplicity, thus avoids many logistical issues and likely increases patients’ willingness to participate in the study.

### 4.4. Considerations for Follow-Up

Generally, the effects of nutritional intervention can take time, and are only valuable if they persist. For future studies, we propose a study duration of at least six months, as such durations are associated with success of a nutritional intervention [209]. Notably, this proposed duration is longer than most previous nut intervention trials, which ranged between 3 and 26 weeks [19].

### 4.5. Considerations for the Intervention

Patients allocated to the intervention should consume a certain number of nuts. In line with previous intervention trials, we propose that the daily intake during the intervention should be at least 50 g [210]. These nuts should ideally be distributed to patients in packed sachets with the set number of nuts, to improve homogeneity of the intervention and adherence to the prescribed number of nuts.

There is no consensus on the type of nut that may have most beneficial health effects, and dietary enrichment of mixed nuts appears a sensible choice [21,210]. These mixed nuts could include unsalted raw peanuts, cashew nuts, walnuts, almonds, hazelnuts, macadamia nuts, pistachios, pecan nuts and Brazil nuts. All of these have been studied in the past and have suggested health benefits. Combining nuts provides the most variation in the macro- and micro-nutrients. Moreover, recent preclinical evidence suggests that mixed nuts may enhance the digestibility and antioxidant activity compared to single nuts both in vitro and in vivo [211]. We propose an intervention with unroasted nuts, considering that nut roasting diminishes micro-nutrient content [148].

In the study, participants may be advised to choose one or several set moment(s) of nut intake during the day to improve implementation and adherence. Moreover, they may be advised to avoid post-prandial nut intake, to avoid study participants having to ingest the nuts while they are already full, although recent studies suggest energy compensation regardless of dietary substitution instructions [52]. In addition to these interventions, patients will continue to follow their regular treatment regimen and should not be instructed to make other dietary changes.

### 4.6. Considerations for Outcome Measures

Although exact study endpoints should be carefully determined depending on study population and research aims, we propose that the primary outcome of this pilot study should be to assess the treatment effects on inflammation (e.g., as measured using circulating high-sensitivity C-reactive protein, glycA and interleukin-6) and innate immune function, specifically phagocytic capacity. Secondary outcomes can focus on whether nutritional status and cardiometabolic status were improved as a result of the intervention. This may be quantified by assessing changes in circulating concentrations and 24 h urinary excretion of all mentioned micro-nutrients, and alterations in circulating HDL cholesterol, LDL cholesterol and triglyceride concentrations. Other exploratory study outcomes may include blood pressure, BMI-adjusted waist circumference, waist–hip ratio, blood pressure, NT-proBNP and arterial stiffness. Other factors, including potential changes in the microbiome, fibrosis, circulating mycotoxin concentrations may also be explored, as the extent to which these factors are affected by nut intake remains unclear.

### 4.7. Considerations for Sample Size

**Figure 2:**
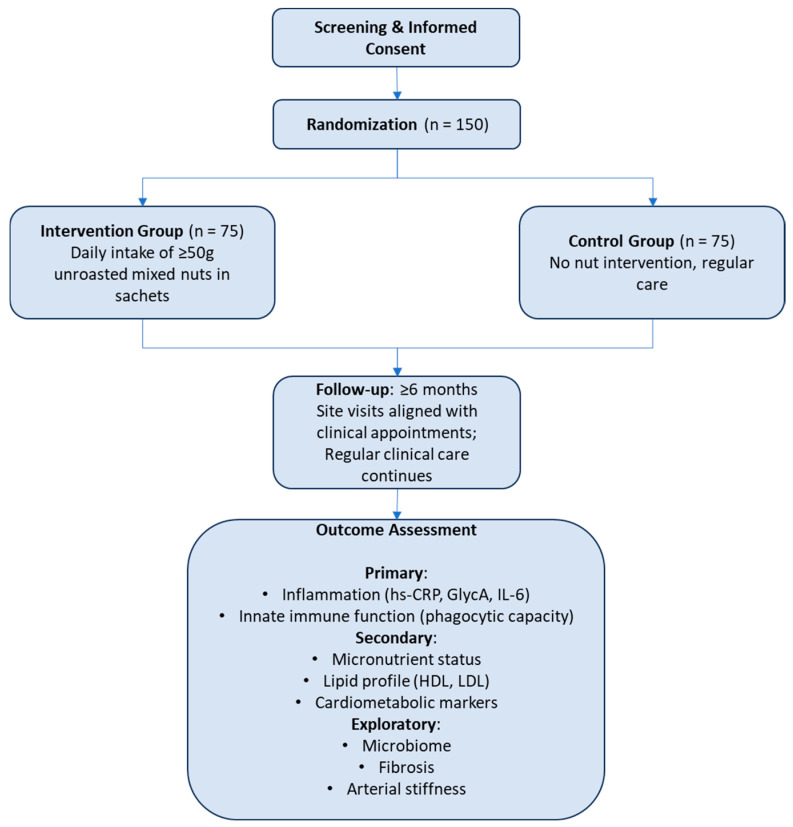
Flow diagram of a parallel randomized controlled trial of mixed nut intervention in kidney transplant recipients.

To our knowledge, the effects of a nut intervention have not been studied in kidney transplant recipients. Therefore, any future study on this topic should be considered a pilot. Sample sizes for pilot studies are generally difficult to assess, and the numerous study outcome measures of interest further complicate any sample size calculations. In line with previous suggestions on sample sizes in pilot studies with continuous outcome variables, we therefore propose a conservatively large sample size of 75 patients per group [212], i.e., 150 patients in total. While indeed, this is a large sample size for a pilot study, this sample size may also allow for sufficient power in post hoc analyses and future hypothesis-generation. An exemplified study design is shown in Figure 2.

## 5. Conclusions

In conclusion, there is a substantial body of evidence supporting the potential beneficial effects of dietary nut enrichment in kidney transplantation. Nuts are rich in minerals, vitamins and fatty acids, and their overall nutritional value may inhibit inflammation and improve cardiometabolic risk. These benefits are particularly relevant for kidney transplant recipients. In the general population, sufficient scientific evidence exists to recommend dietary nut enrichment for the reduction in chronic inflammation and cardiometabolic risk factors. There is a clear need to ultimately translate these observations to dietary recommendations for kidney transplant recipients. Next to a need for more observational and mechanistic studies in kidney transplant recipients regarding the effect of nut consumption on risk factors and health outcomes, we propose that in the future, a randomized, parallel-group trial is needed to investigate the multiple potential beneficial effects of a mixed unsalted nut intervention in this vulnerable patient population.

## Figures and Tables

**Table 1:** Nutrient composition of commonly consumed nuts (adapted from [15] under the CC BY-NC 4.0 license; and [17]; seeds, coconut and chestnut omitted).

Nutrients, per 100 g	Almonds, Unsalted	Brazil Nuts	Cashews, Unsalted	Hazelnuts	Macadamia Nuts	Peanuts, Unsalted	Pecans, Unsalted	Pine Nuts	Pistachio, Unsalted	Walnuts
Energy, kcal (kJ)	607 (2540)	659 (2760)	583 (2440)	628 (2630)	716 (3000)	587 (2460)	697 (2920)	673 (2820)	581 (2430)	654 (2740)
Protein, g	20.3	14.3	14.9	15.0	7.80	24.4	8.9	13.7	20.4	15.2
Carbohydrate, g	20.4	11.7	31.7	16.7	12.8	21.3	13.4	13.1	27.4	13.7
Total sugars, g	4.7	2.3	4.9	4.3	4.1	4.9	3.9	3.6	7.5	2.6
Total dietary fiber, g	10.6	7.5	2.9	9.7	8.0	8.4	9.3	3.7	10.0	6.7
Total fat, g	54.0	67.1	48.0	60.8	76.1	49.7	72.8	68.4	47.4	65.2
Saturated fat, g	4.4	16.1	9.3	4.5	11.9	7.7	6.4	4.9	5.9	6.1
Monounsaturated fat, g	33.3	23.9	27.8	45.7	59.3	26.2	40.8	18.8	25.1	8.9
Polyunsaturated fat, g	13.8	24.4	8.8	7.9	1.5	9.8	22.2	34.1	14.2	47.2
Water, g	2.3	3.4	1.7	5.3	1.6	1.8	3.4	2.3	1.8	4.1
Vitamin A, RAE (μg RAE)	0	0	0	1.0	0	0	3.0	1.0	13.0	1.0
α-Carotene, μg	0	0	0	3.00	0	0	0	0	0	0
β-Carotene, μg	1.0	0	0	11.0	0	0	28.0	17.0	154	12.0
β-Cryptoxanthin, μg	0	0	0	0	0	0	9	0	0	0
Lutein + zeaxanthin, μg	1	0	22	92	0	0	16	9	1125	9
Thiamin, mg	0.075	0.617	0.194	0.643	0.710	0.152	0.640	0.364	0.674	0.341
Riboflavin, mg	1.161	0.035	0.194	0.113	0.087	0.197	0.126	0.227	0.227	0.150
Niacin, mg	3.53	0.30	1.36	1.80	2.28	14.40	1.13	4.39	1.33	1.13
Vitamin B-6, mg	0.13	0.10	0.25	0.56	0.36	0.47	0.20	0.09	1.09	0.54
Total folate, μg	53	22	67	113	10	97	21	34	49	98
Total choline, mg	50.5	28.8	59.2	45.6	44.6	64.6	39.3	55.8	69.3	39.2
Vitamin C, mg	0	0.7	0	6.3	0.7	0	1.1	0.8	2.9	1.3
Vitamin E (α-tocopherol), mg	23.5	5.7	1.2	15.0	0.6	4.9	1.7	9.3	2.5	0.7
Vitamin K, μg	3.5	0	37.2	14.2	0	0	6.9	53.9	16.3	2.7
Calcium, mg	260	160	44	114	70	58	68	16	104	98
Phosphorus, mg	457	725	475	290	198	363	269	575	455	346
Magnesium, mg	271	376	252	163	118	178	117	251	106	158
Iron, mg	3.62	2.43	5.82	4.70	2.65	1.58	2.46	5.53	3.91	2.91
Zinc, mg	3.21	4.06	5.43	2.45	1.29	2.77	4.39	6.45	2.27	3.09
Copper, mg	1.07	1.74	2.15	1.73	0.57	0.43	1.16	1.32	1.25	1.59
Selenium, μg	1.9	1920.0	11.3	2.4	11.7	9.30	3.7	0.7	9.7	4.9
Potassium, mg	692	659	548	680	363	634	398	597	977	441
Sodium, mg	3	3	16	0	353	6	0	2	6	2
SFAs										
6:0, g	0	0	0	0	0	0	0	0	0	0
8:0, g	0.00	0.00	0.13	0.00	0.00	0.00	0.00	0.00	0.00	0.00
10:0, g	0.00	0.00	0.13	0.00	0.00	0.00	0.00	0.00	0.00	0.00
12:0, g	0.00	0.00	0.76	0.00	0.08	0.00	0.00	0.00	0.00	0.00
14:0, g	0.018	0.046	0.337	0.00	0.668	0.016	0.00	0.00	0.012	0.00
16:0, g	3.54	9.63	4.51	3.10	5.93	3.98	4.53	3.21	5.14	4.40
18:0, g	0.78	6.24	2.98	1.27	2.28	1.20	1.79	1.39	0.64	1.66
20:0, g	<0.2	0.3	0.7	<0.2	2.6	1.7	<0.2	0.4	<0.2	<0.2
22:0, g	<0.2	<0.2	<0.2	-	0.7	3.0	-	<0.2	<0.2	<0.2
24:0, g	-	-	<0.2	-	<0.2	1.7	-	-	-	-
MUFAs										
16:1, g	0.26	0.21	0.32	0.12	12.70	0.03	0.01	0.02	0.46	0.00
18:1, g	33.0	23.6	27.2	45.4	44.4	25.4	40.6	17.9	24.4	8.8
20:1, g	0.02	0.03	0.15	0.13	1.93	0.63	0.21	0.80	0.12	0.13
22:1, g	0.000	0.000	0.000	0.000	0.237	0.055	0.000	0.000	0.005	0.000
PUFAs										
18:2, g	13.6	24.4	8.5	7.8	1.3	9.7	21.1	33.2	13.8	38.1
18:3, g	0.162	0.036	0.308	0.087	0.196	0.026	1.110	0.164	0.358	9.080
20:4, g	0.000	0.000	0.000	0.000	0.000	0.016	0.000	0.000	0.005	0.000

Abbreviations: -, not determined; SFA, saturated fatty acids; MUFA, monounsaturated fatty acids; PUFA, polyunsaturated fatty acids; RAE, Retinol Activity Equivalents.

## Data Availability

No new data were created or analyzed in this study. Data sharing is not applicable to this article.
